# Effects of Motor Task Difficulty on Postural Control Complexity during Dual Tasks in Young Adults: A Nonlinear Approach

**DOI:** 10.3390/s23020628

**Published:** 2023-01-05

**Authors:** Marina Saraiva, João Paulo Vilas-Boas, Orlando J. Fernandes, Maria António Castro

**Affiliations:** 1 RoboCorp Laboratory, i2A, Polytechnic Institute of Coimbra, 3046-854 Coimbra, Portugal; 2 Faculty of Sports, University of Porto, 4200-450 Porto, Portugal; 3 LABIOMEP-UP, Faculty of Sports and CIFI2D, University of Porto, 4200-450 Porto, Portugal; 4 Sport and Health Department, School of Health and Human Development, University of Évora, 7000-671 Évora, Portugal; 5 Comprehensive Health Research Center (CHRC), University of Évora, 7000-671 Évora, Portugal; 6 Department of Mechanical Engineering, University of Coimbra, CEMMPRE, 3030-788 Coimbra, Portugal; 7 Sector of Physiotherapy, School of Health Sciences, Polytechnic Institute of Leiria, 2411-901 Leiria, Portugal

**Keywords:** motor dual task, center of pressure, approximate entropy, DFA, correlation dimension, Lyapunov exponent

## Abstract

Few studies have evaluated the effect of a secondary motor task on the standing posture based on nonlinear analysis. However, it is helpful to extract information related to the complexity, stability, and adaptability to the environment of the human postural system. This study aimed to analyze the effect of two motor tasks with different difficulty levels in motor performance complexity on the static standing posture in healthy young adults. Thirty-five healthy participants (23.08 ± 3.92 years) performed a postural single task (ST: keep a quiet standing posture) and two motor dual tasks (DT). i.e., mot-DT(A)—perform the ST while performing simultaneously an easy motor task (taking a smartphone out of a bag, bringing it to the ear, and putting it back in the bag)—and mot-DT(T)—perform the ST while performing a concurrent difficult motor task (typing on the smartphone keyboard). The approximate entropy (ApEn), Lyapunov exponent (LyE), correlation dimension (CoDim), and fractal dimension (detrending fluctuation analysis, DFA) for the mediolateral (ML) and anterior-posterior (AP) center-of-pressure (CoP) displacement were measured with a force plate while performing the tasks. A significant difference was found between the two motor dual tasks in ApEn, DFA, and CoDim-AP (*p* < 0.05). For the ML CoP direction, all nonlinear variables in the study were significantly different (*p* < 0.05) between ST and mot-DT(T), showing impairment in postural control during mot-DT(T) compared to ST. Differences were found across ST and mot-DT(A) in ApEn-AP and DFA (*p* < 0.05). The mot-DT(T) was associated with less effectiveness in postural control, a lower number of degrees of freedom, less complexity and adaptability of the dynamic system than the postural single task and the mot-DT(A).

## 1. Introduction

Many studies use linear measures to assess the center of pressure (CoP) behavior and to characterize postural sway during quiet standing with the aim to analyze changes in postural control during aging or in dual-task conditions [1,2], to evaluate the risk of fall [3], or to study postural control impairments in pathological conditions [4,5], for example. However, the traditional linear characteristics of the center of pressure trajectories can not be sensitive to changes in postural control associated with age or diseases [6]. Thus, a need has emerged for consistent approaches to obtain physiological information from stabilograms using nonlinear approaches to assess CoP temporal time series [7]. Furthermore, nonlinear measures can be more sensitive in detecting postural control impairments than linear measures [8].

Nonlinear measures quantify the regularity, stability, adaptability to the environment, dimensionality, and complexity of the human postural system [9,10,11]. We chose to analyze four nonlinear measures: the approximate entropy (ApEn), the Lyapunov exponent (LyE), the correlation dimension (CoDim), and the detrended fluctuation (DFA, detrended fluctuation analysis) by the scaling exponent (*α*), because these measures reflect the deterministic and stochastic components of motor control (regularity, local stability, number of degrees of freedom, and the presence or absence of correlations in the CoP trajectories).

Approximate entropy is a measure to assess a system’s complexity and the regularity in time-series data. The algorithm of ApEn was introduced by Pincus [12,13,14] to quantify the regularity of biological signals and clinical time series data. Furthermore, some studies used the approximate entropy method for the center of pressure time-series analysis [15,16]. The ApEn values range from 0 to 2; a high ApEn value corresponds to random time series and an increased system complexity with less regular patterns in the time series of the CoP [12,14].

The Lyapunov exponent measures the rate at which nearby orbits converge or diverge in the state space. It has been used to assess the presence of chaos in dynamic systems and analyze various biological systems (e.g., gait, postural sway) [9,17,18,19]. A high LyE value can indicate a faster response capacity of postural control in the face of different perturbations to body movement [7].

The correlation dimension was introduced by Grassberger and Procaccia [20] for calculating the dimensionality of an attractor. It allows evaluating how the data point in a time series of a dynamic system is organized within a state space, in which a small correlation dimension value (between 1.5 and 2.5) can be associated with a small number of degrees of freedom involved and, generally, characterize data of a deterministic nature [17].

Detrended fluctuation analysis is a fractal dimension analysis method for biological time series indicating the presence or absence of correlations in the CoP trajectories by the scaling exponent (*α*). A scaling exponent equal to 0.5 corresponds to white noise (uncorrelated data), *α* equal to 1.0 indicates pink noise, and *α* equal 1.5 indicates Brown noise [21]. The pink noise may be representative of a complex movement (more flexible), the brown noise of a constrained movement, and the white noise of an incoherent movement [17]. Although the DFA can be used to analyze the time series of the CoP trajectories [22], it also has other practical applications, such as in the prediction of type 2 diabetes mellitus [23] and in the analysis of heart rate times series [21].

Although these measures represent different aspects of system dynamics, they are related concepts. Combining them can give researchers different insights into system dynamics and postural stability patterns (see [7] for a recent review).

Maintaining a controlled upright posture is essential to performing various activities of daily living; beyond that, most people stand or walk while performing another task (cognitive or motor secondary task); this is called dual task. When people perform a dual task, there is usually a deterioration in one or both tasks’ performance [24]. For example, some studies showed that walking while simultaneously carrying a cup [25] or transferring coins from one pocket to the other [26] reduces the gait performance compared to only walking. Others studies reported that maintaining an upright position while performing a cognitive task decreases the postural stability compared to performing a single task [27,28].

Currently, a prevalent dual task is the use of smartphone functions while walking or standing. However, smartphone use is associated with sedentary behaviors [29], injuries [30] and sleep disorders [31] and affects the balance ability negatively [32,33,34]. In addition, studies showed that smartphone use while maintaining a standing posture increased postural sway [35,36] and might cause changes in the complexity of the center of pressure during some dual-task conditions [36].

### Study Purpose

To our knowledge, there are few studies evaluating the effect of secondary motor tasks on standing posture (primary motor task) [35,37,38]. Besides, few studies used nonlinear measures to assess postural control during dual-task conditions [39,40,41]. Concerning the effect of smartphone use on postural stability, the dual-task studies’ results are contradictory, and few were based on a nonlinear analysis [42]. Thus, this study aimed to evaluate the effects of two motor secondary tasks with different levels of difficulty on static standing posture, based on CoP nonlinear analysis. We hypothesized that young adults present less effectiveness, complexity, and adaptability of the postural control when performing a difficult motor dual task than a postural single task and easy motor dual task. We conjecture that these nonlinear time series analyses will provide helpful information about secondary motor tasks’ effects on the motor complexity of standing posture performance.

## 2. Materials and Methods

The number of participants in the study was determined using G*power software (Franz Faul, Edgar Erdfelder, Axel Buchner, Universität Kiel, Germany, version 3.1.9.6) based on the study design, with a significance level of α = 0.05, a power of 0.95, and a large effect size (Cohen’s *f* = 0.40). A sample minimum number of 18 individuals was found to be necessary.

The study was publicized on social networks and in groups of friends to recruit young adults between 18 and 35 years interested in participating and fulfilling the eligibility criteria. Thirty-five healthy young adults (22 males and 13 females) were recruited, without cognitive, vestibular, neurological, or musculoskeletal disorders (the sample characteristics are reported in Table 1).

All participants gave prior consent to the experimental procedures in agreement with the Declaration of Helsinki. The data were collected in the Robocorp Laboratory, Polytechnic Institute of Coimbra, and the study was approved by the Ethics Committee of the Polytechnic Institute of Coimbra (approval number: 27_CEPC2/2019).

### 2.1. Task Protocol

**Figure 1:**
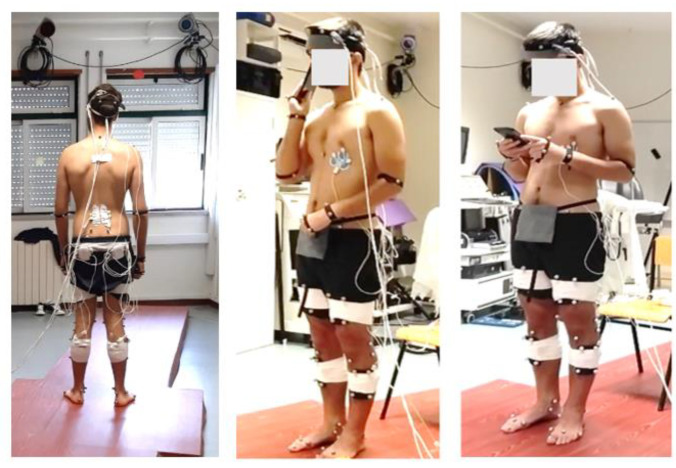
Center-of-pressure time series in the anterior-posterior and mediolateral displacement collected from a force plate during the postural single task and the easy and difficult motor dual tasks, respectively, from left to right.

Each participant performed each task twice for 60 s, with 45 s of rest between each task, i.e., the static standing posture (postural single task) and two motor dual tasks with different challenges while using their smartphone (easy and difficult motor dual tasks) (Figure 1). No priority was given to the secondary motor and standing postural tasks. Instead, the participants were instructed to use their smartphone and hold it as usual while performing the easy and difficult dual tasks.

### 2.2. Postural Single Task (ST)

The participants were instructed to stand comfortably on a force plate with feet shoulder-width apart, eyes open, looking in the forward direction, and with their arms naturally at their sides during 60 s [35,43]. This task is usually used as the baseline in dual-task studies on static postural standing [35,36].

### 2.3. Dual-Task Conditions

Easy motor dual-task (mot-DT(A)). The participants were instructed to perform the postural single task while simultaneously taking their smartphone out of a bag, bringing it to the ear, and putting it back in the bag. All participants had a bag with the same dimensions placed in the middle of the pelvis.

Difficult motor dual task (mot-DT(T)). The participants were instructed to perform the postural single task while simultaneously typing on a smartphone. The participants were informed to type randomly on the smartphone keyboard at a self-selected pace to neutralize or minimize the cognitive component.

### 2.4. Standing Postural Sway Dynamics Analysis

The center-of-pressure time series in the anterior-posterior and mediolateral displacement were collected from a Bertec^®^ force plate computation (model FP4060-07-1000; Bertec Corporation, Columbus, OH, USA).

Four nonlinear measures were considered to evaluate the behavioral features of the postural motor task: approximate entropy, largest Lyapunov exponent, detrending fluctuation analysis, and correlation dimension. The nonlinear measures were calculated for each task using values of embedding dimensions through a Matlab routine (version R2020b, The Mathworks, Inc. US, Natick, MA, USA). The nonlinear measures were calculated for each task using a code through Matlab (version R2020b, The Mathworks, Inc., US). The data time series were calculated as follows. To analyze the ApEn of physiological signals, values of *m* of 2 or 3 and of *r* ranging from 0.1 to 0.3 have been recommended. For the calculation of the approximate entropy of the CoP data, the parameters *m* = 2 and *r* = 0.15 were commonly selected. Given time series data of length (N), the approximate entropy was calculated using a lag value of 20, a pattern length (*m*) of 2, and an error tolerance (r) of 0.2 times the standard deviation of the data file [14,20,44].

The phase space was reconstructed to determine time lag and embedding dimension according to the method of Broomhead [45,46,47]. The state space reconstruction was made for calculating the nonlinear parameters by embedding time lag (τ) copies of the time series. The average mutual information (AMI) was used to calculate τ, and we selected the first minimum of the AMI [17,48]. The embedding dimension or the minimum number of variables required to form a valid state space from a given time series was calculated using the false nearest neighbor (FNN) method, with code from the UNO Biomechanics Laboratory. After finding these two parameters, we used the Wolf algorithm created by the University of Nebraska Omaha (UNO) based on the Wolf’s method [49], to calculate the large Lyapunov exponent. We used the Lyapunov exponent to quantify the chaotic behavior of postural sway, i.e., how the movement trajectories under study were related to each other in time. Positive values greater than zero indicate that the postural control system derives from a process exhibiting chaotic dynamics. The largest Lyapunov exponent and the correlation dimension were calculated using a time lag value of 20 and an embedding dimension of 5. The correlation dimension quantifies the dimensionality of the attractor using the Grassberger and Procaccia method [20], well explained in the Appendix by Gurses and Celik [50].

The detrended fluctuation analysis analyzes the self-similarities between fluctuation patterns across progressively long time series. The DFA assesses the growth rate of detrended root-mean-square (RMS) values over many different measurement time scales. To determine the alfa value (scaling exponent, α1), the code from UNO was used to first integrate the time series and then create a new time series. Second, we calculated the root mean of the new time series. This time series was divided into boxes of equal length, and the best-fitting line segment determined the trend within each box. Finally, the average distance fluctuation F(s) of each point in a time series from a local trend line was estimated at a given scale. This method was introduced by Peng et al. [21] and permits the detection of long-range correlations embedded in a nonstationary time series. The scaling exponent α1, obtained from the slope of the linear regression of F(s) over on a log–log scale, quantifies the long-range correlations in the time series. We used the code from UNO to calculate the values of a scaling component based on some studies (see references [17,21,51,52] for more details).

### 2.5. Statistical Analysis

The analyses were performed using IBM-SPSS 25.0 software. The statistical significance level was set at *p* < 0.05. Descriptive statistics were used to summarize the sample characteristics using mean ± SD (standard deviation).

Homogeneity of variances and normality of the distribution of the parameters was tested with the Levene’s and Shapiro–Wilk tests, respectively. Some outcomes were not normally distributed; thus, the median and interquartile range (IQR) represented the data. The Friedman test was used to compare the differences between the postural single task, mot-DT (A), and motor-DT (T) for each nonlinear parameter with post hoc Bonferroni correction to evaluate pairwise comparisons.

## 3. Results

Most of the examined young adults (97.1%) performed mot-DT (T) (keep a quiet standing position while typing on a smartphone keyboard) with both hands. During mot-DT (A), 85.7% of the participants held their smartphone with the right hand. There were no differences in the nonlinear measures between the participants who held the smartphone with one or both hands (*p* > 0.05).

**Figure 2:**
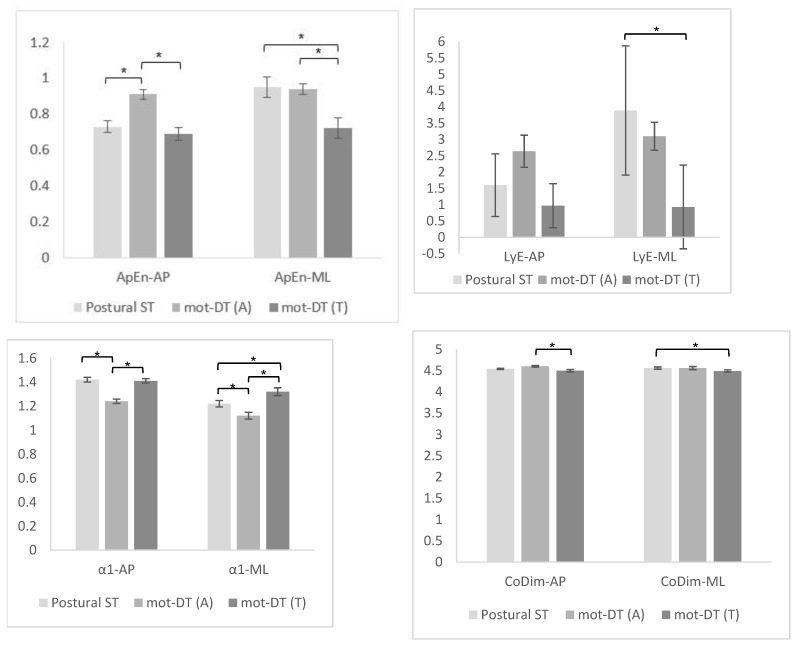
Comparisons between postural single task (ST) and easy and difficult motor dual tasks: CoP nonlinear analysis. ST, single task; Mot-DT (A), easy motor dual task; Mot-DT (T), difficult motor dual task; ApEn, approximate entropy; LyE, Lyapunov exponent; α1, detrending fluctuation analysis (short-term); CoDim, correlation dimension, AP, anterior-posterior; ML, mediolateral. The y−axis displays the median values of the nonlinear measures, and the error bars, the standard error. * *p* < 0.05: Friedman test with Bonferroni correction for multiple comparisons.

The results of approximate entropy, Lyapunov exponent, detrending fluctuation analysis (short-term: α1), and correlation dimension for the postural single task and the dual tasks with different levels of difficulty in anterior-posterior and mediolateral directions are presented in Table 2, and the post hoc analyses in Figure 2.

### 3.1. Approximate Entropy

The results showed a significant difference for ApEn-AP and ApEn-ML between thepostural single task and the dual tasks with two different challenging levels (*p* < 0.001 for anterior-posterior and mediolateral directions). The post hoc analyses showed a significant increase in ApEn-AP during the performance of the easy motor dual task compared to the postural single task (*p* < 0.001) and the difficult motor dual task (*p* < 0.001). However, no differences between postural the single task and the difficult motor dual task were found. The ApEn-ML decreased from the postural single-task to both dual-task conditions; there was a significant difference between the performance of the postural single task and that of the difficult motor dual task (*p* = 0.002) and the performances of the easy and difficult motor dual tasks (*p* = 0.001). However, no performance differences between the postural single task and the easy motor dual task were found.

### 3.2. Lyapunov Exponent

The analysis showed a significant difference in the Lyapunov exponent in the mediolateral direction between the postural single task and the dual tasks with two different challenging levels (*p* = 0.016). However, no differences were found between the three tasks for the anterior-posterior direction.

In LyE-ML, post hoc analyses showed a significant decrease between the postural single task and the difficult motor dual task (*p* = 0.012). However, no differences were found between the easy and the difficult motor dual tasks and between the postural single the task and easy motor dual task.

### 3.3. Detrending Fluctuation Analysis (Short-Term: α1)

The results showed a significant difference for α1-AP (*p* < 0.001) and α1-ML (*p* < 0.001) between postural single task and dual tasks with two different challenging levels.

The post hoc analyses showed a significant increase in α1-AP during the difficult motor dual task compared to the easy motor dual task (*p* < 0.001). There was a significant decrease in α1-AP from the postural single task to the easy motor dual task (*p* < 0.001). However, no differences between postural single task and difficult motor dual task were found. The α1-ML was higher during the difficult motor dual task than the easy motor dual task and the postural single task; these differences were significant (*p* < 0.001 and *p* = 0.004, respectively). There was a significant decrease in α1-ML from the postural single task to the easy motor dual task (*p* = 0.036).

### 3.4. Correlation Dimension

The analysis showed a statistical significance for CoDim-AP (*p* = 0.022) and CoDim-ML (*p* = 0.019) between the postural single task and the dual tasks with two different challenging levels. The post hoc analyses showed a significant decrease in CoDim-AP from the easy motor dual task to the difficult motor dual task (*p* = 0.018) and in CoDim-ML from the postural single task to the difficult motor dual task (*p* = 0.018). However, no differences were found between the postural single task and the easy motor dual task in CoDim anterior-posterior and mediolateral directions. No significant differences were found between the postural single task and the difficult motor dual task in CoDim anterior-posterior direction. Furthermore, no differences were found between the easy and the difficult motor dual tasks in CoDim-ML.

## 4. Discussion

In this study, we used a nonlinear analysis to infer about the complexity of the postural task (standing posture performance) during the performance of dual tasks with different difficulty levels. Based on the CoP nonlinear analysis, our results showed changes in postural control complexity when comparing single-task to dual-task conditions with different challenge levels. The results suggested that performing a difficult motor dual task was associated with less effectiveness in postural control as well as less complexity and adaptability of the dynamic system than performing a postural single task and an easy motor dual task.

Across the postural single task to the difficult motor dual-task, the examined young adults showed a significant decrease in the LyE-ML and ApEn-ML values and an increase in the α1-ML (close to brown noise), suggesting a lower postural control adaptability to external and internal perturbations. The ApEn-ML and α1-ML values followed the same trend for the task performance from the easy to the difficult dual task. These results indicate less postural control in the mediolateral center-of-pressure direction during the difficult dual task than during the postural single task and the easy motor dual task.

Previous studies reported that performing a dual task (standing while performing a cognitive task) was associated with a diminished complexity of postural control compared to performing a single task (quietly standing) in older adults using multiscale entropy analysis [53,54]. A study found higher sample entropy values in the mediolateral direction for each dual-task condition (with different challenging cognitive task levels) than for the single task (quietly standing) in young adults, showing an increase in the efficiency of postural control during the dual tasks [55]. Another study found no differences between standing upright with eyes open while performing a cognitive task (dual task) and perfoming a single task (standing upright with the eyes open) using sample entropy analysis in young adults. However, the authors found an increase in LyE and CoDim during the dual task compared to the single task [56], contradicting our LyE and CoDim results. Furthermore, the use of secondary cognitive tasks while performing a postural task appears to improve the postural control (increased stability) due to automatized postural control [39,57]. The results of these studies may not be the most adequate to explain our results since they use cognitive tasks instead of secondary motor tasks. However, our study also showed that the nonlinear analysis performed allowed us to detect a short-term change in postural control complexity in response to adding a secondary motor task while simultaneously keeping a standing posture.

Contrary to our difficult motor dual-task results, a study that assessed the effect of texting using a mobile phone on the postural stability of young adults using multivariate multiscale entropy analysis found no difference between normal stance (single task) and normal stance with texting (dual task). However, it found differences between conditions with and without texting in tandem stance, showing more complexity in the case of a dual task in tandem stance [36]. An explanation for this could be that the task of texting has a cognitive component (involves reading and typing), and in our study, the young adults only randomly typed on the smartphone keyboard during a normal stance; in addition, the entropy analysis method used was different.

During the difficult motor dual task, the young adults kept their gaze directed towards the smartphone screen, reducing their field of vision (reduced visual input) compared to when performing the postural single task and the easy motor dual task; maybe for that reason, we observed an increase in α1, since a higher α short-term value is associated with decreased center-of-pressure complexity during quiet standing with eyes closed in young adults [58].

Both tasks’ correlation dimension values were high, characterizing completely random data [17]. However, the young adults demonstrated a significant reduction in the correlation dimension in their center-of-pressure data in the mediolateral direction from the postural single task to the difficult motor dual-task. Furthermore, this reduction was also verified from the easy to the difficult motor dual task, but in the anterior-posterior direction. These results can indicate that during the performance of the difficult motor dual task, there was an increased postural control in the mediolateral and anterior-posterior center-of-pressure components compared to the performances of the postural single task and the easy motor dual task, respectively, due to the reduced degrees of freedom involved.

In the anterior-posterior displacement of the center of pressure, a significant decrease in ApEn and an increase in α1 (close to brown noise) were found in young adults performing the difficult dual task compared to when performing the easy motor dual task. These data demonstrated that the young adults presented less complexity and adaptability of the postural control in the anterior-posterior CoP direction during the difficult motor dual task than during the easy motor dual task.

When comparing the performance of the postural single task to that of the easy motor dual task, the results showed a significant decrease in α1 (anterior-posterior and mediolateral directions, close to pink noise) and an increase in ApEn-AP, showing less regularity and more complexity and adaptability of the postural control during the easy motor dual task than the postural single task. During arm raising, anticipatory postural adjustments occur in the direction opposite to the reaction forces caused by the arm movement [59], thus preserving postural control during the perturbation caused by upper limb elevation. Furthermore, the automatic postural responses can be modified by maturation and motor experience [60], and in young adults spending more time using their phones in their daily lives [61,62], the anticipatory postural adjustments during an easy motor dual-task condition are possibly more efficient than those required when performing a postural single task, which may be an explanation for these results. Based on the ApEn analysis, a study also found a higher ApEn value (more random) in the CoP-AP time series during a dual task (performing the sensory organization test while performing a cognitive task) than a single task [16].

A strength of this study is the nonlinear analysis of motor dual-task conditions with different challenge levels, reflecting the characteristics and changes of the complexity and variability of the center-of-pressure displacement during natural daily life tasks. Furthermore, using a dual-task paradigm to evaluate if the nonlinear analysis could detect a short-term change in postural control in response to the addition of a secondary motor task while keeping a standing posture is an innovation.

Our results showed that typing on the smartphone keyboard could be more difficult due to less complexity and adaptability of the postural system during difficult motor dual tasks. Furthermore, the difficult motor dual task implied a closed posture and fine motor movements to manipulate the smartphone and involved more visual monitoring and a reduced field of view than the easy motor dual task. Based on some definitions of and research about task difficulty (for more details, see [63,64,65]), we defined that typing on the smartphone keyboard would be a difficult motor task, whereas taking the smartphone out of a bag, bringing it to the ear, and putting it back in the bag would be an easy task. Although there is not a clear and explicit definition of task difficulty, it also involves the interactions between task, task performer, and task context, referring to the perception of task performers’ difficulty in performing a task [66]. However, we did not ask the participants about their perceived difficulty regarding the tasks. Thus, we consider this a limitation of this study and recommend that future studies assess the perception of difficulty by the task performer pre- and post-task to help define a motor task difficulty level.

Future research should include electromyographic and nonlinear analysis to understand better the maintenance of balance by muscle activation around the ankle joint and the complexity of the center of pressure while performing dual-task. Besides, it would be interesting to analyze other methods of nonlinear analysis in dual-task conditions. For example, the extended detrended fluctuation analysis can be helpful in posturography to identify differences in postural control strategies between healthy and pathological groups while performing everyday tasks [67]. The ApEn algorithm can produce a bias towards regularity when counting self-matches from each subseries [15]; therefore, comparing the results obtained through ApEn with other entropy analysis methods would be relevant.

In addition, we recommended applying this study’s methodology to other age groups, pathological conditions, and postural tasks with different levels of demand to assess the dual-task effect on the dynamic postural system. Finally, future research should also include the study of other behaviors, as well as multitasking.

The present study’s nonlinear results can provide helpful information about the secondary motor tasks’ effects on the motor complexity and adaptability of the dynamic system of CoP during dual-task conditions. Furthermore, the differences in postural control complexity from the single task to the easy and difficult motor dual tasks suggest that motor demands vary in their impact on the postural sway complexity.

The increased motor task demand during dual tasks causes a loss of motor system complexity, showing an increasingly ineffective and inadequate postural control strategy. Therefore, it is essential in clinical practice to implement strategies to improve postural control performance, such as dual-task training using different tasks to enhance the dynamic organization of the center-of-pressure displacements.

## 5. Conclusions

We found changes in postural control complexity from postural single-task to motor dual-task conditions with different difficulty levels using a nonlinear analysis of the center of pressure. Furthermore, our results suggested that performing a difficult motor dual task is associated with less effectiveness in postural control and less complexity and adaptability of the dynamic system of the center-of-pressure displacement than performing apostural single task and an easy motor dual task. For this reason, it is important to implement appropriate clinical practices, such as dual-task training, to improve the postural control complexity under dual-task conditions. We suggest that the nonlinear analysis of the center of pressure be performed in other age groups, pathological conditions, and with postural tasks with different levels of demand to evaluate the effect of dual tasks on the postural system complexity.

## Figures and Tables

**Table 1:** Anthropometric characteristics of the sample (mean ± SD).

Variables	Sample *n* = 35
Age (years)	22.94 ± 3.88
Height (m)	1.71 ± 0.10
Body mass (kg)	73.63 ± 16.06
Body mass index (kg/m^2^)	24.98 ± 4.32

**Table 2:** Comparisons of CoP time series displacements among postural single task and easy and difficult motor dual tasks, median (IQR).

Nonlinear Measures	Single Task	Mot-DT (A)	Mot-DT (T)	*p*-Value ^1^
ApEn-AP	0.73 (0.62–0.91)	0.91 (0.77–1.03)	0.69 (0.57–0.91)	<0.001 *
ApEn-ML	0.95 (0.72–1.20)	0.94 (0.88–1.06)	0.72 (0.49–0.96)	<0.001 *
LyE-AP	1.60 (0.42–6.47)	2.64 (1.00–4.82)	0.97 (0.17–5.61)	0.091
LyE-ML	3.89 (0.93–17.81)	3.10 (1.23–5.77)	0.93 (0.18–8.27)	0.016 *
α1-AP	1.42 (1.30–1.51)	1.24 (1.16–1.34)	1.41 (1.30–1.47)	<0.001 *
α1-ML	1.22 (1.09–1.32)	1.12 (1.03–1.27)	1.32 (1.24–1.51)	<0.001 *
CoDim-AP	4.54 (4.49–4.59)	4.60 (4.51–4.65)	4.50 (4.38–4.60)	0.022 *
CoDim-ML	4.56 (4.49–4.67)	4.56 (4.39–4.66)	4.49 (4.38–4.55)	0.019 *

ST, single task; Mot-DT (A), easy motor dual task—performing the postural single task while simultaneously taking the smartphone out of a bag, bringing it to the ear, and putting it back in the bag; Mot-DT (T), difficult motor dual-task—performing the postural single task while simultaneously typing on the smartphone; ApEn, approximate entropy; LyE, Lyapunov exponent; α1, detrending fluctuation analysis (short-term); CoDim, correlation dimension, AP, anterior-posterior; ML, mediolateral. ^1^ Friedman test (differences between the three tasks); * *p* < 0.05.

## Data Availability

Not applicable.
